# Chromosome-level genome assembly of the bethylid ectoparasitoid wasp *Sclerodermus* sp. ‘alternatusi’

**DOI:** 10.1038/s41597-024-03278-0

**Published:** 2024-05-02

**Authors:** Yi Wan, Hui-Jie Wu, Jia-Peng Yang, Jin-Li Zhang, Zhi-Cheng Shen, Hai-Jun Xu, Yu-Xuan Ye

**Affiliations:** 1 State Key Laboratory of Rice Biology and Breeding, Key Laboratory of Biology of Crop Pathogens and Insects of Zhejiang Province, Institute of Insect Sciences, Zhejiang University, Hangzhou, 310058 China; 2 Zhejiang University Zhongyuan Institute, Zhengzhou, 450000 China

**Keywords:** Next-generation sequencing, Evolutionary genetics

## Abstract

The Bethylidae are the most diverse of Hymenoptera chrysidoid families. As external parasitoids, the bethylids have been widely adopted as biocontrol agents to control insect pests worldwide. Thus far, the genomic information of the family Bethylidae has not been reported yet. In this study, we crystallized into a high-quality chromosome-level genome of ant-like bethylid wasps *Sclerodermus* sp. ‘alternatusi’ (Hymenoptera: Bethylidae) using PacBio sequencing as well as Hi-C technology. The assembled *S. alternatusi* genome was 162.30 Mb in size with a contig N50 size of 3.83 Mb and scaffold N50 size of 11.10 Mb. Totally, 92.85% assembled sequences anchored to 15 pseudo-chromosomes. A total of 10,204 protein-coding genes were annotated, and 23.01 Mb repetitive sequences occupying 14.17% of genome were pinpointed. The BUSCO results showed that 97.9% of the complete core Insecta genes were identified in the genome, while 97.1% in the gene sets. The high-quality genome of *S. alternatusi* will not only provide valuable genomic information, but also show insights into parasitoid wasp evolution and bio-control application in future studies.

## Background & Summary

Parasitic Hymenoptera is hyperdiverse in the insect lineages and comprises a prominent plurality of venomous species^1^. This group have evolved diversified parasitic strategies to manipulate their hosts, such as producing venom^2^, polydnavirus^3^, teratocytes^4^, ovarian proteins^5^, and larval secretions, leading to host killed or paralyzed permanently^6^. Parasitoid wasps provide a sustainable approach in biocontrol of insect pests, thus conferring enormous economic and ecological benefits to global agriculture and forestry^7,8^. For instance, an obligate aphid parasitoid *Aphidius gifuensis* (Hymenoptera: Braconidae) was applied to control the green peach aphid *Myzus persicae*, one of the most economically important aphid crop pests worldwide^9,10^. The pupal endoparasitoid *Trichopria drosophilae* (Hymenoptera: Diapriidae) was used to control the spotted wing drosophila *Drosophila suzukii*, a fruit fly that causes massive economic damage to a variety of summer fruit in United States^11^. Recently, parasitoid wasps are instantly developing as a promising model to gain insight into genome size evolution and parasite-host coevolution^12,13^.

**Fig. 1:**
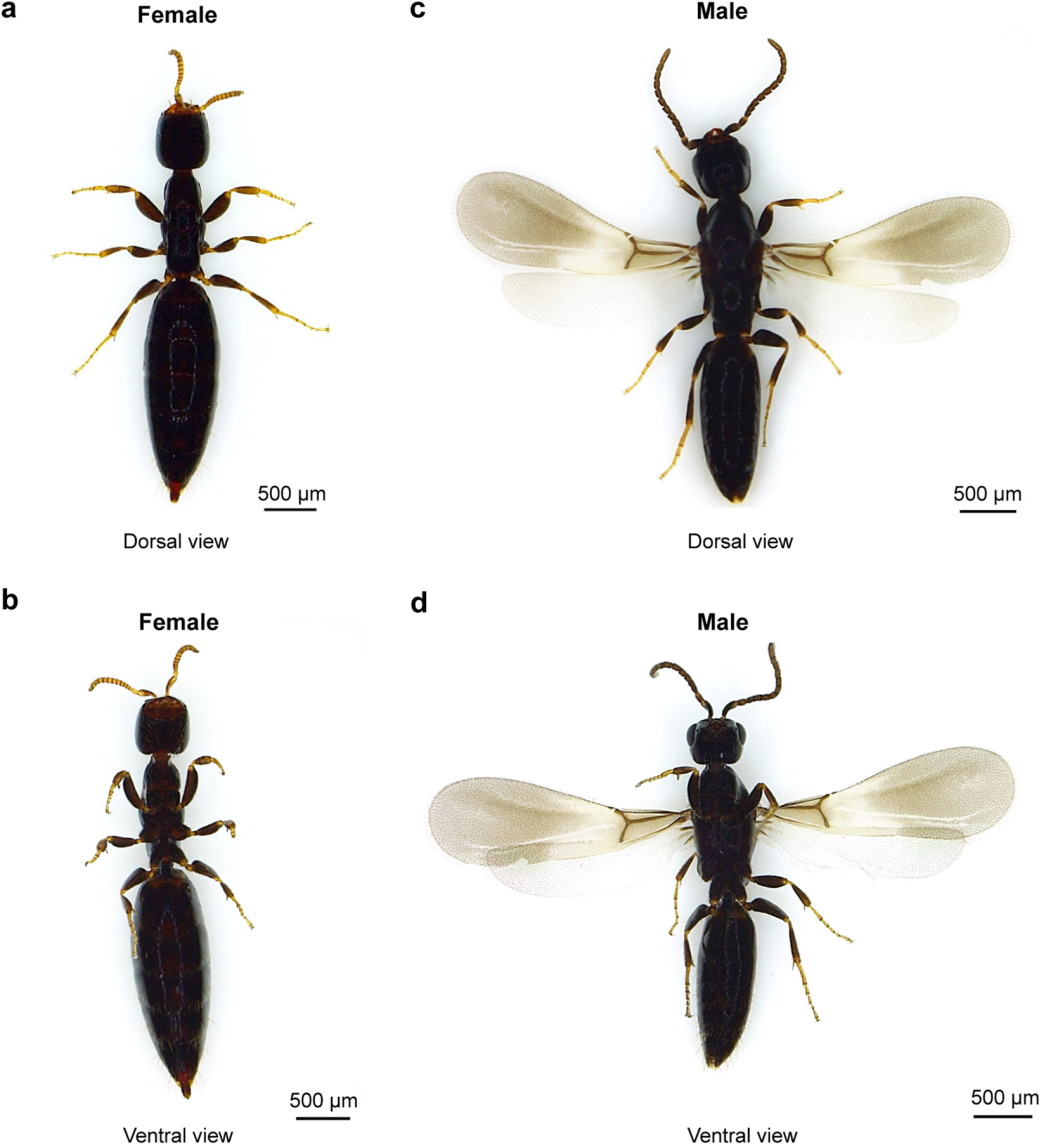
Morphology of *S. alternatusi* adults. (**a**) Dorsal view of a wingless female. (**b**) Ventral view of a wingless female. (**c**) Dorsal view of a winged male. (**d**) Ventral view of a winged male.

**Fig. 2:**
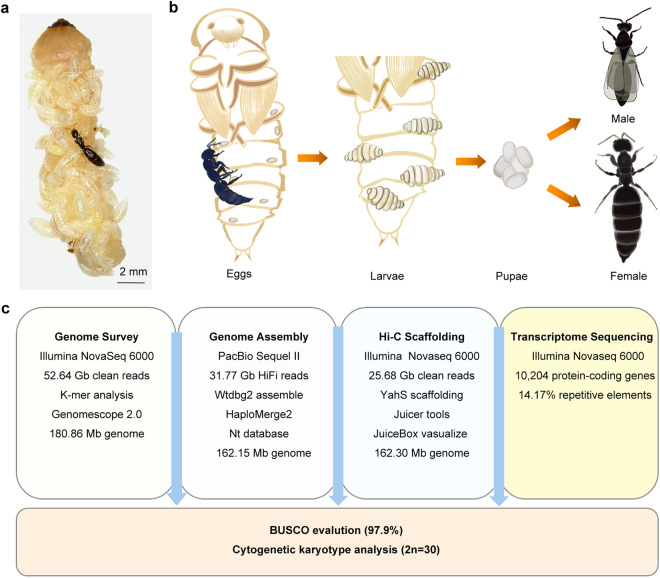
Life cycle of *S. alternatusi* and workflow used in the genome sequencing and assembly. (**a**) A parasitized larva of the longhorn beetle *Thyestilla gebleri*. Both *S. alternatusi* female and larvae are shown. (**b**) Laboratory rearing of *S. alternatusi* using a substitute host *T. molitor* pupae. The winged ratio of males and females is 90% and 50%, respectively. (**c**) The workflow overview of *S. alternatusi* chromosome-level genome assembly.

The Bethylidae are the most diverse of Hymenoptera chrysidoid families, with more than 3,000 external parasitoids of Lepidopteran and Coleopteran larvae^14^. The bethylids have been widely adopted as biocontrol agents to control insect pests worldwide. The ant-like bethylid wasps *Sclerodermus* sp. ‘alternatusi’ and *S. guani* (Hymenoptera: Bethylidae) are ectoparasitoid wasps indigenous to China^15^. The adults *S. alternatusi* are brown-colored insects with stout legs, morphologically resembling *S. guani*. The head is distinctly prognathous and 13 antennomers in both sexes. The eye length of males is a little more than half the length of the head and gibbose, but the eyes of females are reduced. Females tend to be wingless (Fig. 1a,b), whereas males are mostly winged with strongly-reduced veins (Fig. 1c,d). The metasoma has seven or eight abdominal segments externally visible. Both species generally parasitize the larva of wood-boring insects such as *Monochamus alternatus*, which vectors pinewood nematode *Bursaphelenchus xylophilus*, the causal agent of pine wilt disease^16,17^. The wood-boring insects are difficult to control, due to its hidden early life, conceal in their habits (trunk, wood, or seed) and long emergence period of the adults^18^. *S. alternatusi* possesses the ability of invading the chamber structures and detecting host larvae or pupae. Females secrete venom from venom reservoir and inject venom subsequently to permanently paralyze the hosts prior to feeding and oviposition. After that, *S. alternatusi* larvae (Fig. 2a) absorb nutrition from the hosts until adult emergence^19,20^. During this process, females protect the eggs and larvae by moving their location upon the host externally. Because of its high parasitism rate and easy artificial rearing (Fig. 2b), *S*. *alternatusi* has been mass bred on a commercial scale^21^.

High-quality assembled genomes contribute to the molecular mechanisms behind parasitic biology. Generally, Hymenopteran parasitoids possess a particular haplodiploid sex determination system, where males are haploid and females are diploid. As such, Hymenopteran males were commonly used for genome sequencing to simplify the genome assembly and annotation due to the lack of heterozygosity^22^. Thus far, more than 100 parasitoid wasps with their genomes sequenced and assembled have been reported, mainly from the family Micryoidea, Ichneumonoidea, Cynipoidea, Cyanoidea and Orussoidea^23^. Among these, 17 species were sequenced using high-throughput chromosome conformation capture (Hi-C) technology (Table 1). However, the genomic information of the family Bethylidae has not yet been reported.

**Table 1:** Summary of genome assemblies of 17 independent parasitoid wasps.

	Species	Assembly size (Mb)	Chr	Contig N50	Scaffold N50 (Mb)	Protein-coding genes	GC content
1	*Aphidius gifuensis*^9^	156.9	6	3.9 Mb	27.5	12,683	17.0%
2	*Pteromalus puparum*^49^	338.2	5	98.8 Kb	65.8	14,946	40.5%
3	*Aphelinus atriplicis*^50^	340.3	4	5.2 Mb	74.3	N/A	41.0%
4	*Aphelinus certus*^50^	333.5	4	29.5 Kb	54.0	N/A	41.0%
5	*Cotesia chilonis*^51^	189.5	10	1.2 Mb	20.4	14,142	30.4%
6	*Cotesia congregata*^52^	199.2	10	52.1 Kb	20.0	13,989	28.0%
7	*Cotesia glomerata*^53^	288.8	10	N/A	27.8	14,119	30.5%
8	*Venturia canescens*^54^	290.8	11	11.2 Mb	25.0	11,831	39.5%
9	*Theocolax elegans*^55^	662.7	7	1.1 Mb	88.8	23,212	33.5%
10	*Megastigmus duclouxianae*^56^	878.5	5	3.9 Mb	215.6	29,206	34.9%
11	*Megastigmus sabinae*^56^	813.0	5	1.4 Mb	139.2	22,994	34.9%
12	*Anastatus japonicus*^12^	950.9	5	N/A	178.3	27,792	30.6%
13	*Anastatus fulloi*^12^	963.4	5	N/A	160.2	27,168	30.0%
14	*Microplitis manilae*^57^	282.9	11	1.8 Mb	25.2	15,689	31.3%
15	*Nasonia vitripennis*^58^	297.3	6	7.2 Mb	7.2	13,602	41.0%
16	*Microctonus hyperodae*^59^	106.7	12	7.6 Kb	9.4	11,688	29.5%
17	*Microctonus aethiopoides*^59^	129.2	8	27.6 Kb	23.0	12,476	29.4%

To gain insights into the evolution of *S. alternatusi* and the complex relationship between the parasitoid and its hosts, we herein developed a high-quality chromosome-level assembly of the *S. alternatusi* reference genome. We integrated PacBio sequencing and Hi-C technology (Fig. 2c) for genome assembly. The *S. alternatusi* genome size is 162.3 Mb with a contig N50 size of 3.83 Mb and scaffold N50 size of 11.1 Mb (Table 2). Notably, 23,014,663 bp repetitive sequences were identified, occupying 14.17% of the *S. alternatusi* genome size (Table 3). Applying Hi-C scaffolding, we assigned 92.85% bases to 15 pseudo-chromosomes, which was further corroborated by karyotyping analysis (2n = 30). In addition, multiple transcriptome data and homologue protein sequences assisted us in annotating 10,204 protein-coding genes (Table 2). For functional annotation of protein-coding genes, we aligned gene sequences to NR, NT, SwissPro, KOG, eggNOG and InterPro databases, in which 10,027, 6,300, 7,515, 5,997, 9,488 and 9,190 genes were successfully mapped (Table 4), respectively.

**Table 2:** Summary of the *S. alternatusi* genome assembly.

	Statistics
Number of scaffolds	174
Number of contigs	261
Genome scaffolds size (bp)	162,309,288
Genome contig size (bp)	162,291,888
Scaffold N50 (bp)	11,104,532
Contig N50 (bp)	3,826,340
Number of chromosomes	2n = 30
Chromosome/total	92.85%
GC content (%)	42.08
Protein-coding gene	10,204

**Table 3:** Annotation of repeat elements in the *S. alternatusi* genome.

Type	Number of elements	Length (bp)	Percentage (%)
LTRs	2,178	2,310,335	1.42
LINEs	1,600	890,966	0.54
DNAs	13,254	5,033,353	3.10
SINEs	22	1,310	0.01
Non-interspersed repeats	187,694	7,824,709	4.82
Unknown	19,427	7,216,968	4.44
Rolling-circles	503	544,066	0.33
tRNA	35	2,469	0.01
Total repeats	224,713	23,014,663	14.17

Abbreviations: LTRs, long terminal repeats; LINEs, long interspersed nuclear elements; DNAs, DNA elements; SINEs, short interspersed nuclear elements. The total number of bases in a repeat sequence is less than the sum of the bases in each type of repeat sequence due to an overlapping between different types.

**Table 4:** Statistical analysis of the functional gene annotations of the *S. alternatusi* genome.

Database	Number	Percent (%)
NR	10,027	94.69
NT	6,300	59.50
SwissProt	7,515	70.97
KOG	5,997	56.63
eggNOG	9,488	89.60
Interpro	9,190	86.79
Annotated	10,204	96.36
Unannotated	385	3.64

## Methods

### Insect

The parasitoid wasp *S. alternatusi* colony was maintained in our laboratory, Zhejiang University, Hangzhou, China. Wasps were reared on a substitute host, the yellow mealworm beetle *Tenebrio molitor* (Fig. 2b), and maintained at 26 °C and 70% relative humidity in the 5 mL finger-shaped tubes with cotton plugged.

### Genome survey

**Fig. 3:**
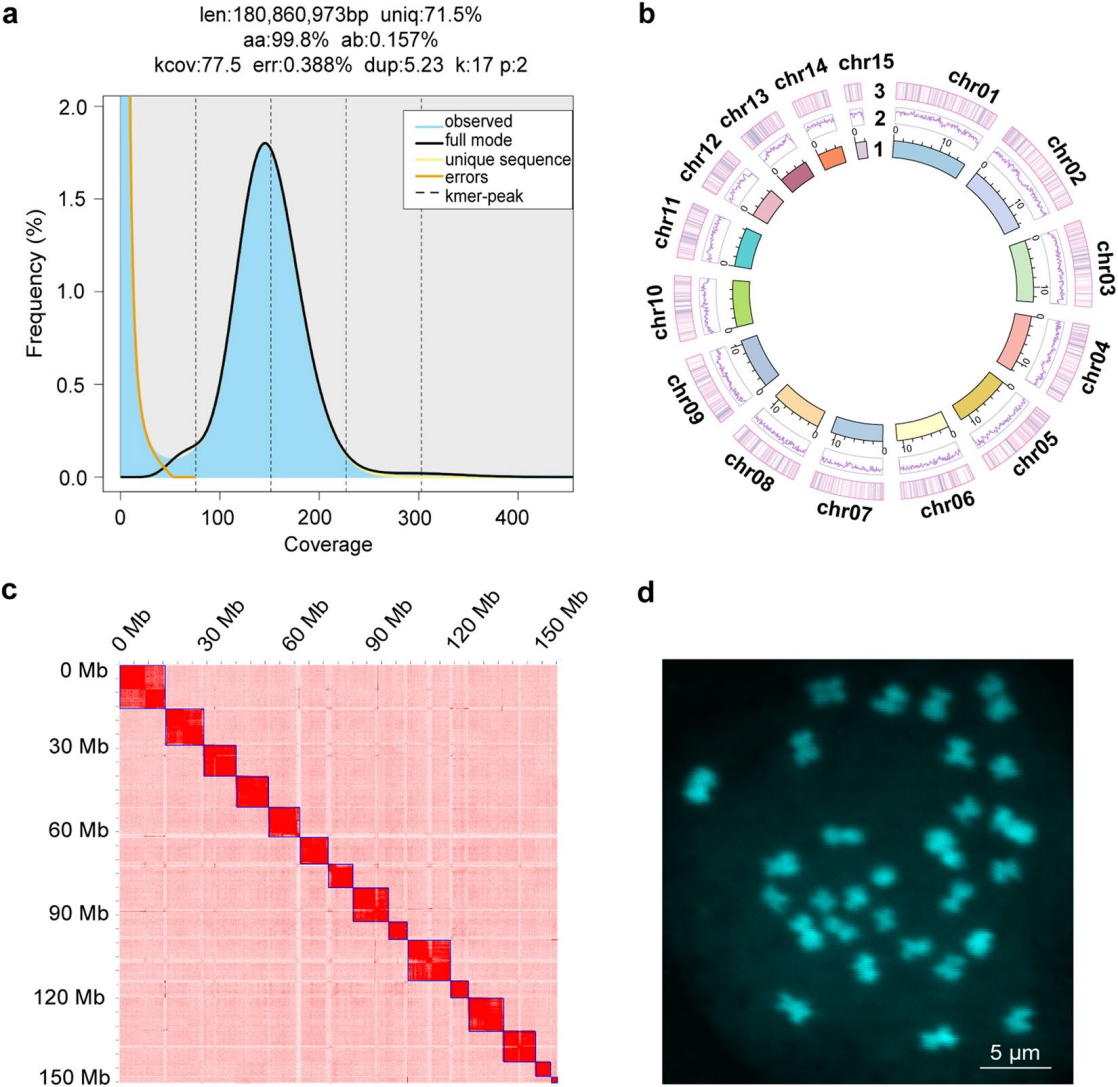
Characteristics of the *S. alternatusi* genome. (**a**) *S. alternatusi* genome size estimation by *K*-mer distribution (*K* = 17). The x-axis represents the *K*-mer depth and the y-axis is the corresponding frequency. (**b**) Circos plot of the *S. alternatusi* genome assembly. Tracks from inner to outer represent the following: (1) 15 pseudo-chromosomes at the Mb scale; (2) GC content; and (3) genes density. (**c**) Hi-C heatmap of *S. alternatusi*. The scale bar shows all interaction frequency of 15 chromosomes. (**d**) Karyogram of *S. alternatusi*: 2n = 30.

Qualified genomic DNA (gDNA) was extracted from 300 female adults. A DNA library was constructed using TruSeq Nano DNA library kit (Illumina, USA), with an average insert size of 350 bp. The library was sequenced using Illumina NovaSeq 6000 platform from Annoroad gene technology Co., Ltd. (Beijing, China). To obtain clean reads, raw data were filtered by removing low quality, short reads, cut adapters, and polyG. In total, 52.64 Gb clean reads were maintained for subsequent survey analysis. The genome size, heterozygosity, and repeat content of *S. alternatusi* were estimated by Genomescope v2.0^24^ (https://github.com/tbenavi1/genomescope2.0), and the results were visualized by *K*-mer (k = 17, produced by Jellyfish v2.2.10) frequency distribution map^25^ (Fig. 3a). The estimated genome size of *S. alternatusi* was 180.86 Mb, with the estimated genome repeat length of 51.56 Mb and the estimated heterozygosity of 0.157%.

### PacBio library construction and *de novo* assembly

gDNA was extracted from 1,000 female adults using FineOut animal tissue DNA kit (Genfine, China). The DNA integrity was assessed by Agilent 4200 bioanalyser (Agilent Technologies, China). Subsequently, Megaruptor 3 (Diagenode, USA) was applied to shear the gDNA, followed by purification using AMPure PB magnetic beads (Pacific Biosciences, USA). Each SMRTbell library was constructed using the PacBio SMRTbell template prep kit 2.0 (Pacific Biosciences, USA). The BluePippin system was utilized for DNA size selection. The genome of the *S. alternatusi* was sequence on PacBio Sequel II platform, and the output data was visualized using SMRTlink v11.0 (PacBio, USA). A total of 31.77 Gb PacBio CCS (HiFi) reads (after error correction from 546.19 Gb raw data) were obtained with an average length of 16.86 kb and an N50 length of 17.73 kb, which was further used for *de novo* assembly. The assembler software Wtdbg2 v2.5^26^ (https://github.com/ruanjue/wtdbg2-xsq-g180m) delivered initial contigs of high-quality assembly from *S. alternatusi* HiFi reads. The genome size of the first assembly was 180,768,149 bp with a contig N50 of 3,562,991 bp and an Insecta BUSCO completeness of 97.8% (95.1% single-copy and 2.7% duplicated genes). Purge Haplotigs v1.1.1^27^ (https://bitbucket.org/mroachawri/purge_haplotigs/src/-a60) combined with HaploMerger2 v3.6^28^ with default parameters were employed to remove heterozygosity from the first assembly. The NCBI non-redundant nucleotide database (NT) was used to identify and eliminate possible contaminating sequences. The pseudo-haplotype assembly was 162,151,154 bp with a contig N50 of 4,324,342 bp and an Insecta BUSCO completeness of 97.8% (95.8% single-copy and 2.0% duplicated genes).

### Hi-C library construction and sequencing

Chromosome contact information was revealed from Hi-C data. A total of 70 *S. alternatusi* larvae were collected for preparing Hi-C libraries according to standard protocols^29^. The samples were crosslinked with 2% formaldehyde solution at room temperature for 10 min and then added with 2.5 M-glycine solution priority to quality control. Hi-C libraries were constructed and sequenced using Illumina Novaseq 6000 platform. Hi-C data were used to anchor the contigs to chromosomes as well as orienting the scaffolds into super scaffolds. We used SAMtools (https://github.com/samtools) and Chromap^30^ with default parameters (https://github.com/haowenz/chromap) to align Hi-C clean reads with the assembled *S. alternatusi* genome. Then YahS^31^ with default parameters (https://github.com/c-zhou/yahs) was used to construct chromosome-scale scaffolds. The contact frequency matrix results were obtained with Juicer tools v1.19.02 (https://github.com/aidenlab/juicer), followed by visualization of contigs and scaffolds using JuiceBox v1.11.08^32^ with default parameters (https://github.com/aidenlab/Juicebox). Ultimately, the size of the *S. alternatusi* genome was 162.30 Mb with a contig N50 of 3.83 Mb (Table 2), and the contigs were anchored to 15 pseudo-chromosomes (Fig. 3b,c, and Table 5).

**Table 5:** Pseudo-chromosome length of *S. alternatusi* by Hi-C.

Pseudomolecule	length (bp)
chr01	15,943,164
chr02	14,698,263
chr03	13,137,865
chr04	12,217,432
chr05	11,878,876
chr06	11,240,777
chr07	11,104,532
chr08	11,025,084
chr09	10,760,464
chr10	9,877,063
chr11	8,381,636
chr12	6,525362
chr13	6,365,108
chr14	5,276,944
chr15	2,277,000

### Cytogenetic karyotype analysis

Heads were dissected from 4–6 d larvae (n = 20) and then mixed with 1 mL of 0.07–0.08 mg/mL colchicine at 25 °C for 3 h. The tissues were treated with 1% hypotonic sodium citrate solution for 1 h, followed by immobilizing with a fixative solution (methanol: acetic acid, 3:1) at 4 °C for 1 h. The tissues were softened in 60% acetic acid for 30 min and then fixed in the fixative solution again for 10 min. Subsequently, samples were ground in the fixative solution using disposable tissue grinding pestles. The cell suspension was dropped onto a pre-chilled glass slide. After being air-dried, the cells were stained with 5 µg/mL DAPI for 5 min and rinsed with running water. Chromosomes (2n = 30) were observed using an Olympus FV3000 microscope with 60 × magnification (Fig. 3d).

### Transcriptome sequencing

For assisting gene annotation, we prepared transcriptomes of *S. alternatusi* from three developmental stages including eggs, pupae (4d, 8d,and 11d), adult females, and adult males. In addition, four representative tissues including head, fat body, ovary and venom glands were dissected from females for transcriptome sequencing. RNAs were extracted using RNAiso Plus (Takara, China) according to the manufacturer’s protocol. An RNA library was constructed using NEBNext ultra RNA library prep kit (NEB, USA) following the manufacturer’s recommendations. RNA sequencing was performed on the Illumina Novaseq. 6000 platform. Full-length transcripts were assembled by Trinity v2.15.1^33^ with default parameters (https://github.com/trinityrnaseq).

### Gene prediction and annotation

A genome-wide annotation tool (GWAT) GETA v2.4.1 with default parameters (https://github.com/chenlianfu/geta) was employed to perform gene prediction and annotation. RepeatMasker v4.1.2 with default parameters (http://repeatmasker.org/RepeatMasker) was applied to identify the repetitive sequences, encompassing interspersed repeats and transposable elements (TEs). Then RepeatModeler v2.0.3 with default parameters (http://www.repeatmasker.org/RepeatModeler) and Repbase library^34^ (https://www.girinst.org/repbase) assisted to cluster the repeats by building a *de novo* repeat library. A total of 23,014,663 bp repetitive sequences were obtained, accounting for 14.17% genome size. Four classes of TEs, including long terminal repeats (LTRs), long interspersed nuclear elements (LINEs), DNA elements (DNAs) and short interspersed nuclear elements (SINEs), comprised 1.42%, 0.54%, 3.10% and 0.01% of the *S. alternatusi* genome, respectively (Table 3).

After masking the repeat sequences, the *S. alternatusi* genome was annotated by integrating evidence including *ab initio* gene predictions, transcripts and protein homologues. Augustus v3.4.0^35^ with default parameters (https://github.com/Gaius-Augustus/Augustus) was applied for *ab initio* gene predictions. Then PASA v2.4.1^36^ with default parameters (https://github.com/PASApipeline) was employed to align the transcriptome data to the genome, followed by homology-based prediction using GeneWise v2.4.1^37^ with default parameters (https://www.ebi.ac.uk/Tools/psa/genewise). EVidenceModeler v1.1.1^38^ with default parameters (https://github.com/EVidenceModeler/EVidenceModeler) was employed to integrate the output from the above approaches to generate a combined annotation model. Furthermore, functional annotation of the predicted protein-coding gene was carried out by searching against the NCBI non-redundant databases (NR) (ftp://ftp.ncbi.nlm.nih.gov/blast/db/FASTA/nr.gz), the nucleotide sequence database (NT) (https://www.ncbi.nlm.nih.gov/nucleotide), SwissProt^39^, eukaryotic orthologous groups (KOG)^40^ as well as InterPro^41^. The genes were also mapped to eggNOG database^42^. Ultimately, a total of 10,204 protein-coding genes in the genome were successfully annotated (Table 4).

## Data Records

All data generated during this study including genome assembly, transcriptome assembly and raw sequencing data were submitted to NCBI. The raw sequencing data have been deposited at NCBI Sequence Read Archive (SRA) under BioProject accession PRJNA1087141 with the accession number SRP495066^43^. This Whole Genome Shotgun project has been deposited at GenBank under the accession JBBEEM000000000. The version described in this paper is version JBBEEM010000000^44^. The whole sequencing dataset and genome assembly reported in this paper have been also deposited in the Genome Sequence Archive (GSA) at the National Genomics Data Center (NGDC)/China National Center for Bioinformation (CNCB) under accession number CRA012526^45^. The genome annotation has been deposited in the Genome Warehouse^46^ in National Genomics Data Center^47^, Beijing Institute of Genomics, Chinese Academy of Sciences/China National Center for Bioinformation, under accession number GWHEQBB00000000.

## Technical Validation

The Hi-C intra-chromosomal contact map with high alignment ratio (92.85%), indicated valid interaction information of 15 pseudo-chromosomes such as homologous contact pattern of chromosomes and translocated regions. The chromosome-level genome assembly quality of *S. alternatusi* was evaluated by performing BUSCO v5.4.6^48^ with default parameters, which presented that 97.9% of BUSCO genes (insecta_db10) were successfully identified in the genome assembly, encompassing complete and single-copy (96%), complete and duplicated (1.9%), fragmented (0.1%) and missing (2%) categories. The gene annotation result was assessed using BUSCO, indicating 1,327 (97.1%) genes were functional annotated. In summary, we provide a high-quality genome with high level of completeness and accuracy.

## Data Availability

The command and pipelines used for data analyses in this study were executed according to corresponding protocols of bioinformatics software. No custom programming or coding was used. The version and parameters have been mentioned in Methods.
